# Antistaphylococcal and Antibiotic Resistance Modulatory Activities of Thirteen Cameroonian Edible Plants against Resistant Phenotypes

**DOI:** 10.1155/2018/1920198

**Published:** 2018-07-03

**Authors:** Brice E. N. Wamba, Armelle T. Mbaveng, Paul Nayim, Joachim K. Dzotam, Ornella J. T. Ngalani, Victor Kuete

**Affiliations:** Department of Biochemistry, Faculty of Science, University of Dschang, Dschang, Cameroon

## Abstract

**Background:**

In this study, 18 methanol extracts from Cameroonian edible plants were tested for their antibacterial activities against 26 strains of *S. aureus*; the role of efflux pumps in the resistance of tested bacteria and the antibiotic resistance-modulating activities against selected multidrug-resistant (MDR) phenotypes were also investigated.

**Methods:**

Broth microdilution assay was used to evaluate the antibacterial activity, the role of efflux pumps, and the antibiotic resistance-modulating effects of plant extracts.

**Results:**

Extracts from *Dacryodes edulis* seeds (DES) and *Dacryodes edulis* bark (DEB) were active against all 26 tested bacterial strains, within the minimal inhibitory concentration (MIC) range of 256–1024 *µ*g/mL. MIC values varied from 64 to 1024 *µ*g/mL against 96.2% of the 26 tested bacteria for *Phaseolus vulgaris* leaves (PVL), 92.3% for *Azadirachta indica* bark (AIB), *Dacryodes edulis* leaves (DEL), and *Ricinodendron heudelotii* leaves (RHL). The lowest MIC value of 64 *µ*g/mL was obtained with the extract from *Cucurbita maxima* beans (CMB) against MRSA4 strain and from *Uapaca guineensis* bark (UGB) against MRSA9 strain. Bacterial efflux pump inhibitor (EPI), carbonyl cyanide *m*-chlorophenyl hydrazone (CCCP), improved the activity of DES and UGB as well as that of extracts from *Hibiscus esculentus* leaves (HEL) and *Uapaca guineensis* leaves (UGL) against resistant *S. aureus* strains. Antibiotic-modulating effects against more than 70% of the *S. aureus* strains tested were obtained when RHL (at MIC/2) was combined with CIP, ERY, and KAN (88.89%), CHL (88.89%), TET (77.78%), and STR (88.89%).

**Conclusion:**

The present study demonstrated that the 13 tested plants had antistaphylococcal effects and that DES, HEL, UGL, and UGB could be used in combination with EPI to combat resistance to *Staphylococcus aureus*. Also, it demonstrated that some studied extracts and mostly RHL could be used as antibiotic resistance modulators to fight against resistant strains of *S. aureus*.

## 1. Background

Bacterial infections caused by *Staphylococcus aureus* are globally responsible for 7–10% of deaths annually [1]. It is the most virulent species of the genus *Staphylococcus* and has emerged as one of the most important human pathogens in the last decades, being one of the main causes of hospital and community infections [2]. This bacterium causes a wide range of clinical infections, ranging from common infections such as skin and soft tissue infections to septicemia, pneumonia, and toxinosis [3, 4]. The fight against *S. aureus* is hindered by the development of resistance of various strains to antibiotics [5–9]. The multidrug resistance (MDR) observed in Gram-positive bacteria is mostly attributed to overexpression of efflux pumps and antibiotics-degrading enzymes. This MDR of *S. aureus* propels the search of new antibacterials with more efficiency and low toxicity. Plant kingdom contains a variety of pharmacologically active secondary metabolites, and some of them have been reported for their antibacterial activities [10, 11]. Their use to combat *S. aureus* antibiotic resistance is an attractive strategy. In regard to the loss of efficacy of several antibiotics and the scarcity of new antibacterial agents, it is also important to search for substances capable of restoring the activity of antibiotics. Antibacterial screenings of African plants have yielded promising results in the past [12–15]. The present study was set up to evaluate the antistaphylococcal potential of 13 Cameroonian food plants: *Azadirachta indica* A. Juss (Meliaceae), *Citrus grandis* (L.) Osbeck (Rutaceae), *Cucurbita maxima* Duch. (Cucurbitaceae), *Dacryodes edulis* (G. Don) H. J. Lam (Burseraceae), *Hibiscus esculentus* L. (Malvaceae), *Ipomoea batatas* (L.) Lam (Convolvulaceae), *Irvingia gabonensis* (Aubry. Lec. ex O. Rorke) Baill. (Irvingiaceae), *Phaseolus vulgaris* L. (Fabaceae), *Ricinodendron heudelotii* (Baill.) Pierre ex Heckel (Euphorbiaceae), *Saccharum officinarum* L. (Poaceae), *Spondias mombin* L. (Anacardiaceae), *Theobroma cacao* L. (Sterculiaceae), and *Uapaca guineensis* Muell. Arg. (Euphorbiaceae). The study was extended on the role of efflux pumps in resistance to some plant extracts as well as the ability of extracts to potentiate the activity of selected antibiotics.

## 2. Materials and Methods

### 2.1. Plant Materials and Extraction

The thirteen edible plants were collected from different localities of Cameroon, namely, Obala (Centre Region), Muyuka (South-West Region), and Dschang (West Region) from March to April 2016. The collected plant samples included the bark of *Azadirachta indica* A. Juss (Meliaceae), leaves of *Citrus grandis* (L.) Osbeck (Rutaceae), beans of *Cucurbita maxima* Duch. (Cucurbitaceae), leaves, bark, and seeds of *Dacryodes edulis* (G. Don) H. J. Lam (Burseraceae), leaves of *Hibiscus esculentus* L. (Malvaceae), beans of *Ipomoea batatas* (L.) Lam (Convolvulaceae), leaves of *Irvingia gabonensis* (Aubry. Lec. ex O. Rorke) Baill. (Irvingiaceae), leaves of *Phaseolus vulgaris* L. (Fabaceae), bark and leaves of *Ricinodendron heudelotii* (Baill.) Pierre ex Heckel (Euphorbiaceae), leaves of *Saccharum officinarum* L. (Poaceae), leaves of *Spondias mombin* L. (Anacardiaceae), leaves and beans of *Theobroma cacao* L. (Sterculiaceae), and leaves and bark of *Uapaca guineensis* Muell. Arg. (Euphorbiaceae). Plants were identified at the National Herbarium in Yaoundé (Cameroon) where the voucher specimens were conserved under the registration numbers (Table 1). The dried and powdered material (100 g) of each plant was macerated in 300 mL of methanol at room temperature for 48 h and then filtered using Whatman filter paper No. 1. The filtrate obtained was concentrated using a rotary evaporator under reduced pressure to obtain the crude methanol extract, which was kept at 4°C until further use.

### 2.2. Chemicals

Eight reference antibiotics (RA) purchased from Sigma-Aldrich (St Quentin Fallavier, France) were tested: ampicillin (AMP), cefepime (CEF), chloramphenicol (CHL), ciprofloxacin (CIP), erythromycin (ERY), kanamycin (KAN), streptomycin (STR), and tetracycline (TET); *p*-iodonitrotetrazolium chloride (INT) (Sigma-Aldrich) was used as bacterial growth revelator; carbonyl cyanide *m*-chlorophenyl hydrazone (CCCP) and chlorpromazine (CPZ) (Sigma-Aldrich) were used as efflux pump inhibitors (EPIs); and dimethylsulfoxide (DMSO) was used to dissolve the plant extracts.

### 2.3. Bacteria, Culture Media, and Growth Conditions

The strains of *Staphylococcus aureus* used included a reference strain obtained from American Type Culture Collection (ATCC; ATCC 25923), 8 methicillin-resistant *S. aureus* (MRSA) strains (MSSA1, MRSA3, MRSA4, MRSA6, MRSA8, MRSA9, MRSA11, and MRSA12) (obtained from the culture collection of the Laboratory of Microbiology, Graduate School of Pharmaceutical Sciences, The University of Tokyo, Japan, and provided by Dr Jean P. Dzoyem, University of Dschang) [8, 9], and 17 resistant clinical laboratory strains of *S. aureus* (SA01, SA07, SA18, SA23, SA36, SA39, SA56, SA64, SA68, SA88, SA114, SA116, SA124, SA126, SA127, SA135, and SA139) available in our laboratory collection and previously isolated from patients in Ad-Lucem Hospital in Banka-Bafang (West Region of Cameroon) [54]. Their bacterial features are summarized in Table 2. They were cultured at 37°C overnight on Mueller Hinton agar 24 h prior to any assay. The Mueller Hinton broth (MHB) was used as liquid culture medium for susceptibility tests.

### 2.4. Preliminary Phytochemical Screenings

Potential classes of potential antibacterial phytochemicals such as alkaloids (Dragendorff's and Mayer's tests: 5 mg plant extract in 10 mL methanol; a portion of 2 mL extract + 1% HCl + steam, 1 mL filtrate + 6 drops of Mayor's reagents/Wagner's reagent/Dragendorff's reagent; cream precipitate/brownish-red precipitate/orange precipitate indicated the presence of respective alkaloids), saponins (frothing test: 0.5 mL filtrate+ 5 mL distilled water; frothing persistence indicated the presence of saponins), steroids and triterpenoids (Liebermann–Burchard test: 5 mg plant extract in 10 mL chloroform, filtered; a 2 mL filtrate + 2 mL acetic anhydride+ conc. H_2_SO_4_; blue-green ring or pink-purple indicated the presence of steroids or triterpenoids), phenolics: anthraquinones (5 mg plant extract in 10 mL methanol; a portion of 2 mL + 2 mL ether-chloroform 1 : 1 v/v + 4 mL NaOH 10% (w/v); red color indicated the presence of anthraquinones), flavonoids (5 mg plant extract in 10 mL methanol; a portion of 2 mL + conc. HCl + magnesium; ribbon pink-tomato red color indicated the presence of flavonoids), polyphenols (ferric chloride test: 5 mg plant material in 10 mL methanol; a portion of 2 mL + 2 mL FeCl_3_; violet-blue or greenish color indicated the presence of phenols), and tannins (5 mg plant extract in 10 mL distilled water; a portion of 2 mL + 2 mL FeCl_3_; blue-black precipitate indicated the presence of tannins) (Table 3) were investigated according to the described phytochemical methods [11, 55].

### 2.5. INT Colorimetric Assay for MIC and Minimal Bactericidal Concentration (MBC) Determinations

The MIC and minimal bactericidal concentration (MBC) determinations on various strains of *S. aureus* were performed using the rapid INT colorimetric assay [56] with some modifications as previously described [14, 38]. The samples were dissolved in DMSO/MHB. The final concentration of DMSO was lower than 2.5%. The twofold dilutions of the samples were made in a 96-well microplate, and the tested bacterial concentration was 1.5 × 10^6^ colony-forming unit (CFU)/mL. The microplates were incubated at 37°C for 18 h. All assays were performed in triplicate and repeated thrice. Wells containing MHB, 100 *µ*L of inoculum, and DMSO to a final concentration of 2.5% served as negative control. The MIC of each sample was detected after 18 h incubation at 37°C, following addition (40 *µ*L) of 0.2 mg/mL of INT and incubation at 37°C for 30 minutes as the lowest sample concentration that prevented the color change of the medium and exhibited complete inhibition of microbial growth [56]. The MBC was determined by adding 50 *µ*L aliquots of the preparations, which did not show any growth after incubation during MIC assays, to 150 *µ*L of MHB. These preparations were further incubated at 37°C for 48 h. The MBC was regarded as the lowest concentration of samples, which did not produce a color change after addition of INT as mentioned above [57, 58].

### 2.6. Evaluation of the Role of Efflux Pumps in the Resistance of Selected Bacteria

To evaluate the involvement of efflux pumps in the resistance of selected bacterial strains to some of the active plant extracts, *Dacryodes edulis* seeds (DES), *Hibiscus esculentus* leaves (HEL), *Uapaca guineensis* leaves (UGL), *Uapaca guineensis* bark (UGB), and CIP (reference drug) were tested in the absence or presence of EPI (CCCP (0.5 *µ*g/mL) or CPZ (25 *µ*g/mL)). MICs of samples alone or in combination with EPI were determined as above, and the increase in activity was determined as the ratio of MIC of sample alone versus sample in combination with EPI. All assays were performed in triplicate and repeated thrice.

### 2.7. Antibiotic Activity Modulation Assays

To evaluate the antibiotic resistance modulation activity of the most active extracts: *Azadirachta indica* bark (AIB), *Dacryodes edulis* seeds (DES), *Dacryodes edulis* bark (DEB), *Dacryodes edulis* leaves (DEL), *Phaseolus vulgaris* leaves (PVL), *Ricinodendron heudelotii* leaves (RHL), and *Uapaca guineensis* bark (UGB), a preliminary assay was performed in order to assess the MICs of antibiotics in the absence and presence of these extracts using the broth microdilution method as previously described [14, 38, 56]. *S. aureus* SA88 was used for the preliminary assay, and the samples were tested at various subinhibitory concentrations (MIC/2, MIC/4, MIC/8, and MIC/16). Results allowed to select DEB, DEL, DES, RHL, and UGB to be tested further against *S. aureus* ATCC 25923, and 8 resistant strains of *S. aureus* (MRSA3, MRSA4, MRSA9, MRSA11, MRSA12, SA18, SA36, and SA64) at MIC/2 and MIC/4. Briefly, after serial dilutions of antibiotics, extract was added to each well at its subinhibitory concentrations, the bacterial inoculation was done, and the MIC was determined. Rows receiving antibiotic dilutions without extracts were used for the determination of the MICs of the antibiotics. The modulation factor was defined as the ratio of the MIC of antibiotic alone versus that of antibiotic in the presence of extract. Modulation factor ≥2 was set as the cutoff for biologically significance of antibiotic resistance-modulating effects [59].

## 3. Results

### 3.1. Phytochemical Composition of Plant Extracts

The major classes of phytochemicals were screened in the 18 studied plant extracts (Table 3). It appears that all extracts contained polyphenols and tannins. Other classes of phytochemicals were selectively present. Only the extract of the beans of *Theobroma cacao* contained all the investigated classes of secondary metabolites.

### 3.2. Antibacterial Activity

The antibacterial activities of the 18 tested extracts and ciprofloxacin against 26 strains of *S. aureus* are summarized in Table 4. It appears that extracts from *Dacryodes edulis* seeds (DES) and *Dacryodes edulis* bark (DEB), within a MIC range of 256–1024 *µ*g/mL, and ciprofloxacin (MIC below 4 *µ*g/mL), were active against all 26 tested bacterial strains. Other extracts were selectively active, and MIC values varied from 64 to 1024 *µ*g/mL against 25/26 (96.2%) tested bacteria for *Phaseolus vulgaris* leaves (PVL), 24/26 (92.3%) for *Azadirachta indica* bark (AIB), *Dacryodes edulis* leaves (DEL), and *Ricinodendron heudelotii* leaves (RHL), 23/26 (88.5%) for *Hibiscus esculentus* leaves (HEL), 19/26 (73.1%) for *Uapaca guineensis* leaves (UGL), 18/26 (69.2%) for *Ricinodendron heudelotii* bark (RHB) and *Uapaca guineensis* bark (UGB), 17/26 (61.5%) for *Saccharum officinarum* leaves (SOL), 16/26 (61.5%) for *Ipomoea batatas* leaves (IBL) and *Theobroma cacao* leaves (TCL), 15/26 (57.7%) for *Citrus grandis* leaves (CGL), 14/26 (53.8%) for *Theobroma cacao* beans (TCB), 12/26 (46.2%) for *Cucurbita maxima* beans (CMB), 10/26 (38.5%) for *Spondias mombin* leaves (SML), and 7/26 (26.9%) for *Irvingia gabonensis* beans (IGB). The lowest MIC value of 64 *µ*g/mL was obtained with CMB against MRSA4 strain and UGB against MRSA9 strain.

### 3.3. Role of Efflux Pumps in the Resistance of Strains of *S. aureus*

Four extracts (DES, HEL, UGL, and UGB) and CIP (reference drug) were tested in the absence or presence of CCCP (0.5 *µ*g/mL) and CPZ (25 *µ*g/mL) to evaluate the role of efflux pumps in the resistance of 14 tested *S. aureus* strains. The results are summarized in Tables 5 and 6. It appears that CCCP significantly improved the activity of the 4 tested extracts against the majority of *S. aureus* strains (Table 5). The increase of activity in the presence of CCCP ranged from 2-fold to >128-fold. The highest increase of activity (>128-fold) was obtained when DES, UGB, and UGL were tested in the presence of CCCP on at least one *S. aureus* strain. In contrast, in the presence of CPZ, no improvement in the activity of the four extracts was observed (Table 6). This is clear indication that CCCP was the appropriate EPI of the studied *S. aureus* strains.

### 3.4. Antibiotic Resistance Modulation Activity of Extracts

Seven plant extracts, AIB, DES, DEB, DEL, PVL, RHL, and UGB, at their various subinhibitory concentrations (MIC/2, MIC/4, MIC/8, and MIC/16) were first tested in combination with 8 antibiotics: CHL, TET, CIP, AMP, CEF, ERY, STR, and KAN against *S. aureus* SA88 strain. The results summarized in Table S1 (Supplementary Materials) show that better modulation of the activity of antibiotics was obtained with all extracts at MIC/2 and MIC/4. At their MIC/2, 2-fold or more increase of antibiotic activities was obtained with PVL, AIB, DEB, DES, DEL, UGB, and RHL and 2, 4, 4, 6, 6, 7, and 8 of the 8 tested antibiotics, respectively (Table S1; Supplementary Materials). Consequently, the most active extracts, DEB, DEL, DES, RHL, and UGB, were further tested in combination with the above 8 antibiotics against the reference strains (ATCC 25923) and 8 resistant strains of *S. aureus* at MIC/2 and MIC/4 (Tables S2–S6; Supplementary Materials). Results showed that 2-fold or more antibiotic-modulating effects against more than 70% of the *S. aureus* strains tested were obtained when DEB was combined with CHL at MIC/2 (77.78%; Table S2; Supplementary Materials), when DEL was combined with CHL and STR at MIC/2 (77.78%; Table S3; Supplementary Materials), when DES was combined with CIP (77.78% at MIC/2), CHL (100% and 88.89 at MIC/2 and MIC/4 resp.), TET (77.78% at MIC/2 and MIC/4), and STR (88.89% and 77.78% at MIC/2 and MIC/4 resp.) (Table S4; Supplementary Materials), when RHL was combined with CIP, ERY, and KAN (88.89% and 77.78% at MIC/2 and MIC/4 resp.), CHL (88.89% at MIC/2 and MIC/4), TET (77.78% at MIC/2 and MIC/4), and STR (88.89% at MIC/2) (Table S5; Supplementary Materials), and when UGB was combined with CHL, KAN, and STR (77.78% at MIC/2 and MIC/4) (Table S6; Supplementary Materials).

## 4. Discussion

### 4.1. Phytochemical Composition of Extracts

Polyphenols and tannins were detected in all extracts. The role of several molecules belonging to polyphenols as antibacterials has been demonstrated [10, 11, 38, 61]. Tannins also belong to a class of polyphenols, and its presence in all extracts could in part explain the fact that all the tested extracts were active in at least one strain of the tested bacteria [10]. However, it should be made clear that the presence of a class of secondary metabolite with reported antibacterial effect is not a guarantee of the good activity of a plant. The antibacterial effect depends on the structure and the amount of a particular phytochemical in the plant or possible interactions with other compounds. This could explain why the extract from the beans of *Theobroma cacao* that contained all the investigated classes of secondary metabolites (Table 3) was not the most active sample (Table 4).

### 4.2. Antibacterial Potential of Extracts

Resistance of bacteria to antibiotics propels the search of new agents to fight against MDR phenotypes. In the present study, clinical strains of *S. aureus* used were previously reported as resistant to at least one commonly used antibiotic [8, 9] (Table 2). Several locally isolated strains of *S. aureus* [54] were used herein, to better adapt the study to our environment. According to established criteria, MIC values in the range of 100–1000 *µ*g/mL are indication that plant extracts bear antimicrobial activities [62]. Also, the antibacterial activity of botanicals is considered significant if MIC values are below 100 *µ*g/mL, moderate if 100 ≤ MICs ≤ 625 *µ*g/mL, and weak if MICs > 625 *µ*g/mL [37, 60]. On these bases, it can be deduced that all the tested plant extracts had antistaphylococcal activities, except *Irvingia gabonensis* beans (IGB), with MICs above 1000 *µ*g/mL against all tested strains of *S. aureus* (Table 4). This activity was significant for CMB against MRSA4 strain and UGB against MRSA9 strain (MIC: 64 *µ*g/mL). Most of the recorded MIC values ranged from 512 to 1024 *µ*g/mL, indicating that extracts rather exhibited moderate to low antistaphylococcal effects. However, this activity could be considered important because the clinical strains of *S. aureus* used were resistant phenotypes while extracts were from edible plants. In effect, it was suggested that if botanicals are food plants, as they are allegedly nontoxic or less toxic than other medicinal plants, their antibacterial activity is significant in a range of 100 ≤ MIC ≤ 512 *µ*g/mL and moderately active in a range of 512 < MIC ≤ 2048 *µ*g/mL [63].

### 4.3. Role of Efflux Pumps in Susceptibility of *S. aureus* Strains to the Extracts

Bacterial efflux systems are associated with major human health concerns as they are involved in the resistance of pathogenic bacteria such as *S. aureus* [64–66]. Previously, inhibition of efflux pumps by natural products has been found to improve the activity of antibiotics against *S. aureus*. For example, inhibition of the TetK efflux pump was reported with the essential oil of *Chenopodium ambrosioides* and its constituent *α*-terpinene against *S. aureus* IS-58 strain [66]. In the present study, two well-known EPIs (CCCP and CPZ) were used to assess the implication of efflux pumps in the resistance of the studied *S. aureus* strains to plant extracts. CCCP is an inhibitor of the proton-motive force of ATP-binding cassette (ABC) transporters of several Gram-negative and Gram-positive bacteria, including *S. aureus* [5–7]. CPZ is capable of reversing or reducing the antibiotic resistance of bacteria including *S. aureus* [67], due to its indirect effects on ATPase activity that is dependent upon Ca^2+^ [68]. In the present study, it was found that CCCP contrary to CPZ improved the activity of the four extracts (DES, HEL, UGL, and UGB) (Tables 5 and 6). This indicates that ABC transporters are involved in the resistance of the studied strains of *S. aureus* and that combination of extracts such as HEL, UGL, and UGB with an inhibitor of ABC transporters could improve the antistaphylococcal fight.

### 4.4. Antibiotic Modulation Effects of Extracts

The antibiotic resistance-modulating effects of several botanicals and phytochemicals against resistant bacteria have been documented [12–15, 59, 69]. It has been suggested that extracts capable of potentiating the activity of antibiotics on more than 70% of bacteria could be potential efflux pump inhibitors [70]. In this study, antibiotic modulation activity of extracts at their MIC/2 on more than 70% tested strains of *S. aureus* was obtained with the association of DEB and DEL and with 1/8 (12.5%) tested antibiotics (Tables S2 and S3; Supplementary Materials), UGB with 2/8 (25%) antibiotics (Table S6; Supplementary Materials), DES with 4/8 (50%) antibiotics (Table S4; Supplementary Materials), and RHL with 6/8 (75%) antibiotics (Table S5; Supplementary Materials). Hence, the tested extracts and mostly RHL may act as efflux pump inhibitors [70]. The use of CCCP indicated that ABC transporters were the efflux pumps involved in the resistance of the tested bacteria, suggesting that the above extract could be the inhibitors of such pumps. The potential of the *R. heudelotii* leaf extract (RHL) to reverse antibiotic resistance in Gram-negative MDR bacteria was previously reported [18]. The present study therefore provides more information about the ability of this plant to modulate the activity of antibiotics against resistant strains of *S. aureus*.

## 5. Conclusion

In conclusion, the present work provides informative data about the antistaphylococcal potential of 13 Cameroonian food plants. It also indicates that some extracts such as DES, HEL, UGL, and UGB could be used in combination with EPI to combat resistance of *Staphylococcus aureus* to antibiotics. Finally, this study also demonstrates that some studied extracts and mostly RHL could be used as antibiotic resistance modulators, providing a new weapon against the resistance of *S. aureus* to antibiotics.

## Figures and Tables

**Table 1:** Information on the studied plants.

Species (family); voucher number	Traditional uses	Bioactive or potentially bioactive components	Known antimicrobial activities of plants
*Azadirachta indica* A. Juss (Meliaceae); 4447/SRFK	Antimalarial, anticancer, anti-inflammatory, antidiabetic, antihyperglycaemic, antiulcerous [16]	Alkaloids, glycosides, flavonoids, and saponins [17]	Antibacterial activity of ethanol leaf extract: *Ec*, *Kp*, *Pm*, *Sa*, *Pa*, *Ef* [18]; ethanol and methanol extract of leaves: *Ec*, *Pa*, *St*, *Sa*, *Bp* [17]

*Citrus grandis* (L.) Osbeck (Rutaceae); 25,860/SRFC	Cancer [19, 20]	Alkaloids, cardioglycosides, saponins, tannins, terpenoids, flavonoids, and steroids [21]	Antibacterial activity of petroleum ether, ethyl acetate, chloroform, ethanol, and leaf water: *Ec*, *Sa*, *Pa*, *Pm* [21]

*Cucurbita maxima* Duch. (Cucurbitaceae); 42,449/HNC	Diabetes, cancer, antihypertensive, anti-inflammatory, immunomodulating, and bacterial infections [22, 23]	Tannins, saponins, alkaloids [24], cucurbitaxanthin, gibberellin, and *α*-tocopherol [25]	Antibacterial activity of aqueous seed extract: *Ec*, *Sa*, *Kp*, *Ef*, *Pa* [24], ethanol and aqueous extract of flowers: *St*, *Ec*, *Ef*, *Bc* [26]

*Dacryodes edulis* (G. Don) H. J. Lam (Burseraceae); 1874/SRFK	Gastrointestinal disorders, toothache, earache [27], dysentery, anemia, leprosy, [28], skin diseases, and sickle cell disease [29]	Ethyl gallate and quercitrin [27]	Antibacterial activity of hydromethanolic, butanol, aqueous, ethanolic extract, ethyl acetate of bark: *Ec*, *Pa*, *Bc*, *Sa* [27]

*Hibiscus esculentus* L. (Malvaceae); 42,823/HNC	Inflammation, pain [30], cancer, hypoglycemic [31]	Not reported	Not reported

*Ipomoea batatas* (L.) Lam (Convolvulaceae); 55,594/HNC	Antidiabetic, anti-inflammatory, reducing risk of cardiovascular disease, anticancer, reducing stomach stress, nausea, diarrhea [32]	Caffeoylquinic acid [33], vitamin E, beta-carotene, lutein, saponins [32], flavonoids, and chitinases [34]	Not reported

*Irvingia gabonensis* (Aubry. Lec. ex O. Rorke) Baill. (Irvingiaceae); 52,936/HNC	Diarrhea, hernia, yellow fever, dysentery, antipoison [35, 36], gonorrhea, gastrointestinal and hepatic disorders, wound infections, diabetes, analgesic [37]	Saponins, tannins, phenols, and phlobatannins [35], alkaloids, cardiac glycosides, anthraquinones, tannins, flavonoids [36], 3-friedelanone; betulinic acid; oleanolic acid; 3,3,4-tri-*O*-methylellagic acid; 3,4-di-*O*-methylellagic acid; hardwickiic acid [38]	Antibacterial activity of aqueous and ethanol extract of leaves and bark: *Sa*, *Ec* [36]; S: *Bst*, *Ca*, *Cf*, *Ea*, *Ecl*, *Mm*, *Ng*, *Pa*, *Pm*, *Pv*, *Sa*, *Sd*; *Bc*, *Bm*, *Bs*, *Ck*, *Ec*, *Kp*, *Sfl*, *St*, *Sf* [38]

*Phaseolus vulgaris* L. (Fabaceae); 42,587/HNC	Antioxidant [39], cancer, estrogenic, antidepressant [40], bacterial infections, tuberculosis [41]	Alkaloids, steroids, and flavonoids [42]	Antibacterial activity of aqueous, alcohol, chloroform, ether extract of seeds: *Sa*, *Pa*, *Sf*, *Bs*, *Kp*, *Ec* [41]

*Ricinodendron heudelotii* (Baill.) Pierre ex Heckel (Euphorbiaceae); 50,852/HNC	Cough, antidote, intestinal diseases [43], yellow fever, malaria, stomach pain, headache, dysentery [44]	Tannins, polyphenols, alkaloids, glycosides, flavonoids, steroids, and saponins [43], aleuritolic acid, labda-8(17), 13-diem-3*β*, 15-diol, *E*-ferulic acid octacosylate [44]	Antibacterial activity of methanol extract of the leaves: *Sa*, *Sf*, *Pv*, *Cf*, *Mm*, *Ko*, *Kp*, *Ec*, *Pa*, *St* [43], *Pf*, *Bs*, *Sa*, *Ec*, *Ca*, *Af* [44], *Ec*, *Ea*, *Pa*, *Ps*, *Kp*, *Ecl* [18]

*Saccharum officinarum* L. (Poaceae); 42,958/HNC	Jaundice and liver problems, hemorrhoids, dysentery, menorrhagia [45]	Flavonoids, saponins, tannins, and alkaloids [46]	Antibacterial activity of methanol extract of the stems: *Ec*, *Kp*, *Sa*, *Pa* [46]

*Spondias mombin* L. (Anacardiaceae); 21,249/SRFK	Diuretic, febrifuge, diarrheal diseases, dysentery, hemorrhoids, gonorrhea, leucorrhea [47]	Saponins, tannins, flavonoids, alkaloids, and glycosides [48]	Antibacterial activity of ethanol, methanol, water, and acetone extracts of the leaves: *Kp*, *Sa*, *St*, *Ea*, *Sm* [48]

*Theobroma cacao* L. (Sterculiaceae); 60,111/HNC	Cardiovascular, gastrointestinal, and nervous diseases [49]	Alkaloids, anthraquinones, cardiac glycosides, and saponins [50]	Antibacterial activity: *Sa*, *Ec*, *Sd*, *Kp*, *Sm*, *Pa*, *Pm* [51]
*Uapaca guineensis* Muell. Arg. (Euphorbiaceae); 53,136/HNC	Leprosy, epilepsy, edema, rheumatism, aphrodisiac, fever, inflammation, absorption [52]	Steroids, alkaloid, terpenoids, and gallic acid [53]	Not reported

HNC: Herbier National du Cameroun; SRFC: Société des Réserves Forestières du Cameroun; SRFK: Société des Réserves forestières du Kamerun; Af: *Aspergillus flavus*; Bp: *Bacillus pumilus*; Bc: *Bacillus cereus*; Bm: *Bacillus megaterium*; Bs: *Bacillus subtilis*; Ca: *Candida albicans*; Cf; *Citrobacter freundii*; Ck: *Candida krusei*; Ea: *Enterobacter aerogenes*; Ec: *Escherichia coli*; Ecl: *Enterobacter cloacae*; Ef: *Enterococcus faecium*; Kp: *Klebsiella pneumoniae*; Ko: *Klebsiella oxytoca*; Mm: *Morganella morganii*; Ng: *Neisseria gonorrhoeae*; Pa: *Pseudomonas aeruginosa*; Pf: *Pseudomonas fluorescens*; Pm: *Proteus mirabilis*; Ps: *Providencia stuartii*; Pv: *Proteus vulgaris*; Sa: *Staphylococcus aureus*; Sd: *Shigella dysenteriae*; Sf; *Streptococcus faecalis*; Sf l: *Shigella flexneri*; Sm: *Serratia marcescens*; St: *Salmonella typhi*.

**Table 2:** Bacterial strains and features.

Bacteria	Features	References
ATCC 25923	Reference strain	—
*S. aureus* MSSA1	Clinical isolate: Met susceptible; Nis^r^, Chl^r^	[8, 9]
*S. aureus* MRSA3	Clinical isolate: Ofxa^r^, Kan^r^, Tet^r^, Erm^r^	[8]
*S. aureus* MRSA4	Clinical isolate: Ofxa^r^, Kan^r^, Cyp^r^, Chl^r^, Gen^r^, Nis^r^, Amp^r^	[8, 9]
*S. aureus* MRSA6	Clinical isolate: Ofxa^r^, Flx^r^, Kan^r^, Tet^r^, Cyp^r^, IM/Cs^r^, Chl^r^, Gen^r^, Nis^r^, Amp^r^	[8, 9]
*S. aureus* MRSA8	Clinical isolate: Ofxa^r^, Flx^r^, Kan^r^, Erm^r^, Cyp^r^, Im/Cs^r^, Chl^r^, Gen^r^, Nis^r^, Amp^r^	[8, 9]
*S. aureus* MRSA9	Clinical isolate: Ofxa^r^, Flx^r^, Tet^r^, Erm^r^, Cyp^r^, Im/Cs^r^, Chl^r^, Gen^r^, Nis^r^, Amp^r^	[8, 9]
*S. aureus* MRSA11	Clinical isolate: Ofxa^r^, Kan^r^, Erm^r^, Cyp^r^, Im/Cs^r^, Chl^r^, Nis^r^, Amp^r^	[8, 9]
*S. aureus* MRSA12	Clinical isolate: Ofxa^r^, Flx^r^, Kan^r^, Erm^r^, Im/Cs^r^, Chl^r^, Gen^r^, Nis^r^, Amp^r^	[8, 9]
SA01	Clinical isolate: Erm^r^, Amp^r^	[54]
SA07	Clinical isolate: Erm^r^, Dox^r^	[54]
SA18	Clinical isolate: Amp^r^, Dox^r^, Vm^r^	[54]
SA23	Clinical isolate: Imi^r^, Aug^r^	[54]
SA36	Clinical isolate: Dox^r^, Vm^r^	[54]
SA39	Clinical isolate: Amp^r^	[54]
SA56	Clinical isolate: Amp^r^, Dox^r^	[54]
SA64	Clinical isolate: Amp^r^, Dox^r^	[54]
SA68	Clinical isolate: Amp^r^, Vm^r^	[54]
SA88	Clinical isolate: Erm^r^, Vm^r^	[54]
SA114	Clinical isolate: Amp^r^, Dox^r^	[54]
SA116	Clinical isolate: Erm^r^	[54]
SA124	Clinical isolate: Erm^r^	[54]
SA126	Clinical isolate: Amp^r^, Dox^r^	[54]
SA127	Clinical isolate: Amp^r^, Dox^r^	[54]
SA135	Clinical isolate: Erm^r^	[54]
SA139	Clinical isolate: Erm^r^	[54]

Chl^r^, Cyp^r^, Erm^r^, Flx^r^, Im/Cs^r^, Kan^r^, Met^r^, Ofxa^r^, Tet^r^, Vm^r^, Amp^r^, Dox^r^, Aug^r^, Gen^r^, and Nis^r^ resistance to chloramphenicol, ciprofloxacin, erythromycin, flomoxef, imipenem/cilastatin sodium, kanamycin, methicillin, ofloxacin, tetracycline, vancomycin, ampicillin, doxycycline, augmentin, gentamicin, and nisin, respectively, SA: *Staphylococcus aureus*.

**Table 3:** Extraction yields and phytochemical composition of the plant extracts.

Plant extract and part used		Yields (%)	Alkaloids	Polyphenols	Flavonoids	Anthraquinones	Tannins	Triterpenes	Steroids	Saponins
*Azadirachta indica*	Bark	10.3	+	+	−	−	+	−	+	+
*Citrus grandis*	Leaves	2.6	+	+	−	−	+	+	+	−
*Cucurbita maxima*	Beans	2.6	−	+	−	−	+	+	+	+
*Dacryodes edulis*	Leaves	6.2	−	+	+	+	+	+	+	+
	Bark	9.1	−	+	−	+	+	+	+	+
	Seeds	6.9	−	+	+	+	+	+	+	+
*Hibiscus esculentus*	Leaves	1.9	−	+	−	−	+	−	+	−
*Ipomoea batatas*	Beans	3.3	+	+	+	+	+	+	−	+
*Irvingia gabonensis*	Leaves	6.7	−	+	−	−	+	−	+	+
*Phaseolus vulgaris*	Leaves	1.2	−	+	−	−	+	−	+	+
*Ricinodendron heudelotii*	Bark	2.9	−	+	+	+	+	+	+	−
	Leaves	7.2	−	+	+	+	+	+	+	+
*Spondias mombin*	Leaves	21.4	−	+	−	−	+	+	+	−
*Saccharum officinarum*	Leaves	8.4	−	+	−	−	+	−	+	+
*Theobroma cacao*	Leaves	3.1	−	+	−	−	+	+	+	+
	Beans	6.2	+	+	+	+	+	+	+	+
*Uapaca guineensis*	Leaves	7.3	−	+	−	−	+	+	+	+
	Bark	6.1	+	+	−	−	+	+	+	+

−: absent; +: present; yield calculated as the ratio of the mass of the obtained methanol extract/mass of the plant powder.

**Table 4:** MIC and MBC of the plant extracts and ciprofloxacin against *Staphylococcus aureus* strains.

Bacterial strains^a^	Samples^b^, MIC and MBC in *μ*g/mL (in parentheses)																		
	Plant extracts																		Antibiotic
	AIB	CGL	CMB	DES	DEB	DEL	HEL	IBL	IGB	PVL	RHB	RHL	SML	SOL	TCB	TCL	UGL	UGB	CIP
ATCC 25923	1024 (–)	–	–	1024 (–)	512 (–)	1024 (–)	–	–	–	512 (–)	1024 (–)	512 (–)	–	1024 (–)	–	–	–	–	<0.5 (16)
SA01	512 (–)	1024 (–)	–	512 (–)	512 (–)	512 (–)	512 (–)	–	–	512 (–)	–	512 (–)	–	1024 (–)	512 (–)	–	512 (–)	512 (–)	<0.5 (4)
SA07	1024 (–)	–	–	512 (–)	512 (–)	512 (–)	512 (–)	1024 (–)	–	1024 (–)	–	512 (–)	–	1024 (–)	–	–	512 (–)	–	<0.5 (1)
SA18	512 (1024)	1024 (–)	–	512 (–)	512 (–)	512 (–)	512 (–)	–	1024 (–)	512 (–)	1024 (–)	512 (–)	–	512 (–)	512 (–)	–	512 (–)	1024 (–)	<0.5 (8)
SA23	1024 (–)	512 (–)	–	512 (–)	512 (–)	1024 (–)	512 (–)	1024 (–)	1024 (–)	512 (–)	1024 (–)	512 (–)	1024 (–)	1024 (–)	512 (–)	512 (–)	512 (–)	512 (–)	<0.5 (<0.5)
SA36	512 (–)	–	1024 (–)	256 (1024)	1024 (–)	1024 (–)	512 (–)	1024 (–)	–	1024 (–)	512 (–)	512 (–)	1024 (–)	1024 (–)	1024 (–)	–	512 (–)	512 (–)	1 (8)
SA39	1024 (–)	1024 (–)	–	512 (–)	1024 (–)	1024 (–)	1024 (–)	–	–	1024 (–)	1024 (–)	–	1024 (–)	–	–	512 (–)	512 (–)	512 (–)	<0.5 (16)
SA56	1024 (–)	–	–	1024 (–)	1024 (–)	1024 (–)	1024 (–)	–	1024 (–)	512 (–)	–	512 (–)	–	1024 (–)	–	–	–	1024 (–)	<0.5 (4)
SA64	512 (–)	512 (–)	1024 (–)	512 (–)	1024 (–)	512 (–)	512 (–)	1024 (–)	–	512 (–)	256 (–)	1024 (–)	1024 (–)	–	–	1024 (–)	512 (–)	1024 (–)	4 (8)
SA68	1024 (–)	–	–	1024 (–)	1024 (–)	1024 (–)	1024 (–)	1024 (–)	–	1024 (–)	1024 (–)	512 (–)	–	–	1024 (–)	–	–	–	<0.5 (<0.5)
SA88	512 (–)	1024 (–)	1024 (–)	512 (–)	1024 (–)	1024 (–)	512 (–)	1024 (–)	–	512 (1024)	–	512 (–)	–	1024 (–)	1024 (–)	1024 (–)	1024 (–)	1024 (–)	<0.5 (2)
SA114	1024 (–)	–	–	1024 (–)	1024 (–)	1024 (–)	256 (–)	512 (–)	–	512 (–)	512 (–)	–	1024 (–)	1024 (–)	–	512 (–)	512 (–)	512 (–)	<0.5 (<0.5)
SA116	–	1024 (–)	–	512 (–)	1024 (–)	512 (–)	512 (–)	–	1024 (–)	1024 (–)	–	1024 (–)	1024 (–)	1024 (–)	–	–	512 (–)	–	<0.5 (<0.5)
SA124	–	1024 (–)	–	512 (–)	1024 (–)	–	–	1024 (–)	–	–	–	1024 (–)	–	–	–	512 (–)	–	–	<0.5 (<0.5)
SA126	1024 (–)	–	1024 (–)	512 (–)	512 (–)	1024 (–)	512 (–)	1024 (–)	–	1024 (–)	1024 (–)	1024 (–)	–	–	–	1024 (–)	1024 (–)	–	<0.5 (<0.5)
SA127	512 (–)	–	–	1024 (–)	1024 (–)	1024 (–)	512 (–)	1024 (–)	–	512 (1024)	–	1024 (–)	1024 (–)	–	–	–	–	–	<0.5 (<0.5)
SA135	1024 (–)	–	–	512 (1024)	1024 (–)	1024 (–)	1024 (–)	–	–	1024 (–)	–	512 (–)	–	1024 (–)	–	–	–	–	<0.5 (1)
SA139	512 (–)	–	–	512 (–)	512 (–)	–	1024 (–)	1024 (–)	–	512 (–)	1024 (–)	512 (–)	–	1024 (–)	–	1024 (–)	–	1024 (–)	<0.5 (<0.5)
MSSA1	512 (–)	–	256 (512)	512 (1024)	1024 (–)	256 (–)	512 (1024)	1024 (–)	–	512 (–)	256 (1024)	512 (1024)	–	1024 (–)	256 (–)	512 (–)	256 (1024)	128 (1024)	2 (16)
MRSA3	512 (1024)	1024 (–)	256 (–)	512 (1024)	1024 (–)	512 (–)	1024 (–)	–	1024 (–)	512 (–)	256 (–)	1024 (–)	512 (–)	–	256 (–)	256 (–)	256 (–)	128 (–)	2 (16)
MRSA4	512 (–)	512 (–)	**64** (512)	512 (512)	512 (1024)	256 (–)	1024 (–)	1024 (–)	–	128 (–)	128 (–)	1024 (–)	1024 (–)	1024 (–)	256 (–)	512 (–)	256 (–)	256 (–)	1 (16)
MRSA6	512 (–)	512 (–)	128	512 (–)	1024 (–)	1024 (–)	–	512 (–)	–	1024 (–)	256 (–)	256 (–)	–	–	128 (–)	256 (–)	256 (–)	128 (–)	2 (8)
MRSA8	512 (–)	1024 (–)	128 (1024)	256 (–)	256 (–)	512 (–)	512 (1024)	–	–	512 (–)	256 (–)	512 (–)	–	512 (–)	128 (–)	512 (–)	256 (–)	128 (–)	2 (8)
MRSA9	512 (–)	1024 (–)	128	512 (–)	512 (–)	1024 (–)	512 (–)	256 (–)	1024 (–)	512 (–)	256 (–)	512 (–)	256 (–)	512 (–)	128 (–)	512 (–)	128 (–)	**64** (–)	2 (16)
MRSA11	1024 (–)	1024 (–)	256	512 (–)	512 (–)	1024 (–)	512 (–)	1024 (–)	1024 (–)	512 (–)	256 (–)	512 (–)	–	–	128 (–)	512 (–)	256 (–)	128 (–)	2 (16)
MRSA12	512 (–)	1024 (–)	128	512 (–)	256 (–)	256 (–)	512 (–)	–	–	1024 (–)	256 (–)	512 (–)	–	1024 (–)	128 (–)	512 (–)	256 (–)	128 (–)	2 (4)

^a^Bacterial strain (SA: *Staphylococcus aureus*; MRSA: methicillin-resistant *Staphylococcus aureus*), ^b^samples (AIB: *Azadirachta indica* bark, CGL: *Citrus grandis*, CMB: *Cucurbita maxima* beans, DES: *Dacryodes edulis* seeds, DEB: *Dacryodes edulis* bark, DEL: *Dacryodes edulis* leaves, HEL: *Hibiscus esculentus* leaves, IBL: *Ipomoea batatas* leaves, IGB: *Irvingia gabonensis* beans, PVL: *Phaseolus vulgaris* leaves, RHL: *Ricinodendron heudelotii* leaves, RHB: *Ricinodendron heudelotii* bark, SML: *Spondias mombin* leaves, SOL: *Saccharum officinarum* leaves, TCB: *Theobroma cacao* beans, TCL: *Theobroma cacao* leaves, UGL: *Uapaca guineensis* leaves, UGB: *Uapaca guineensis* bark, and CIP: ciprofloxacin); R: MBC/MIC; (–): >1024(MIC); nc, not calculated; MIC: minimal inhibitory concentration; MBC: minimal bactericidal concentration; CIP: ciprofloxacin; –: MIC and MBC at up to 1024 *μ*g/mL; MIC in bold: significant activity [37, 60].

**Table 5:** MIC of extracts and ciprofloxacin in the absence (−) and presence (+) of carbonyl cyanide *m*-chlorophenyl hydrazone (CCCP) against selected strains of *Staphylococcus aureus*.

Bacterial strains^a^	Samples^b^ and MIC in *µ*g/mL and fold increase of activity (in parentheses)									
	DES		HEL		UGB		UGL		CIP	
	−	+	−	+	−	+	−	+	−	+
ATCC 25923	256	32 **(32)**	256	<8 **(>32)**	–	<8 **(>128)**	1024	<8 **(>128)**	2	1**(2)**
MRSA3	256	<8 **(>32)**	256	<8 **(>32)**	512	<8 **(>64)**	512	<8 **(>64)**	1	<0.5 **(>2)**
MRSA4	256	16 **(16)**	128	16 **(8)**	1024	128 **(8)**	1024	64 **(16)**	2	<0.5 **(>4)**
MRSA6	128	<8 **(>16)**	256	<8 **(>32)**	1024	<8 **(>128)**	512	<8 **(>64)**	1	<0.5 **(>2)**
MRSA8	256	128 **(2)**	128	128 (1)	–	512 **(>2)**	512	128 **(>4)**	<0.5	<0.5 (nd)
MRSA9	128	<8 **(>16)**	64	<8 **(>8)**	1024	<8 **(>128)**	256	<8 **(>32)**	<0.5	<0.5 (nd)
MRSA11	256	<8 **(>32)**	128	<8 **(>16)**	1024	<8 **(>128)**	256	<8 **(>32)**	1	1(1)
MRSA12	256	<8 **(>32)**	128	<8 **(>16)**	1024	<8 **(>128)**	256	<8 **(>32)**	<0.5	<0.5 (nd)
SA01	128	<8 **(>16)**	128	<8 **(>16)**	1024	<8 **(>128)**	256	<8 **(>32)**	<0.5	<0.5 (nd)
SA07	512	<8 **(>64)**	512	512 (1)	512	512 (1)	256	<8 **(>32)**	<0.5	<0.5 (nd)
SA18	1024	128 **(8)**	256	128 **(2)**	1024	512 **(2)**	512	<8 **(>64)**	1	<0.5 **(>2)**
SA88	512	512 (1)	–	–	1024	1024 (1)	1024	256 **(>4)**	1	<0.5 **(>2)**
SA114	1024	<8 **(>128)**	–	–	1024	128 **(8)**	512	<8 **(>64)**	<0.5	<0.5 **(>2)**
SA135	512	64 **(8)**	–	–	–	128 **(>8)**	512	<8 **(>64)**	1	<0.5 **(>2)**

^a^Bacterial strain (SA: *Staphylococcus aureus*; MRSA: methicillin-resistant *Staphylococcus aureus*), ^b^samples (DES: *Dacryodes edulis* seeds, HEL: *Hibiscus esculentus* leaves, UGL: *Uapaca guineensis* leaves, UGB: *Uapaca guineensis* bark, and CIP: ciprofloxacin); MIC: minimal inhibitory concentration; CCCP was tested at 0.5 *µ*g/mL; (–): >1024 *µ*g/mL; values in bold represent increase of activity ≥2.

**Table 6:** MIC of extracts and ciprofloxacin in the absence (−) and presence (+) of chlorpromazine (CPZ) against selected strains of *Staphylococcus aureus*.

Bacterial strains^a^	Samples^b^ and MIC in *µ*g/mL and fold increase of activity (in parentheses)									
	DES		HEL		UGB		UGL		CIP	
	−	+	−	+	−	+	−	+	−	+
ATCC 25923	512	512 (1)	−	−	512	512 (1)	128	1024 (0.13)	2	2 (1)
MRSA3	256	256 (1)	1024	1024 (1)	512	512 (1)	−	−	4	4 (1)
MRSA4	512	512 (1)	−	−	256	256 (1)	1024	1024 (1)	2	2 (1)
MRSA6	256	256 (1)	512	128 (2)	512	512 (1)	256	−(0.25)	2	2 (1)
MRSA8	256	256 (1)	1024	1024 (1)	256	256 (1)	−	−	1	2 (0.5)
MRSA9	256	256 (1)	1024	1024 (1)	512	512 (1)	256	−(0.25)	1	2 (0.5)
MRSA11	512	512 (1)	1024	1024 (1)	512	512 (1)	128	−(<0.13)	4	4 (1)
MRSA12	512	512 (1)	−	−	512	512 (1)	−	−	2	2 (1)
SA01	512	512 (1)	1024	1024 (1)	512	512 (1)	512	512 (1)	1	1 (1)
SA07	512	512 (1)	1024	1024 (1)	512	512 (1)	1024	1024 (1)	4	4 (1)
SA18	512	512 (1)	−	−	1024	1024 (1)	−	−	1	1 (1)
SA88	512	512 (1)	−	−	1024	1024 (1)	−	−	4	4 (1)
SA114	1024	1024 (1)	−	−	1024	1024 (1)	−	−	1	1 (1)
SA135	512	512 (1)	−	−	1024	1024 (1)	−	−	1	1 (1)

^a^Bacterial strain (SA: *Staphylococcus aureus*; MRSA: methicillin-resistant *Staphylococcus aureus*); ^b^samples (DES: *Dacryodes edulis* seeds, HEL: *Hibiscus esculentus* leaves, UGL: *Uapaca guineensis* leaves, UGB: *Uapaca guineensis* bark, CIP: ciprofloxacin); CPZ: chlorpromazine at 25 *µ*g/mL; CIP: ciprofloxacin; MIC: minimal inhibitory concentration.

## Data Availability

The data used to support the findings of this study are included within the article.

## Supplementary Materials

Supplementary Materials Preliminary evaluation of antibiotic resistance modulatory activity of extracts against *S. aureus* SA88 (Table S1); antibiotic resistance modulatory activity of the bark methanol extract from *Dacryodes edulis* bark (DEB) (Table S2); the leaf methanol extract from *Dacryodes edulis* leaves (DEL) (Table S3); the seed methanol extract from *Dacryodes edulis* seeds (DES) (Table S4); the leaf methanol extract from *Ricinodendron heudelotii* leaves (RHL) (Table S5); and the bark methanol extract from *Uapaca guineensis* bark (UGB) (Table S6) at their MIC/2 and MIC/4 on selected strains of *Staphylococcus aureus*.

## Abbreviations

AIB: *Azadirachta indica* bark
AMP: Ampicillin
ATCC: American Type Culture Collection
CCCP: Carbonyl cyanide *m*-chlorophenyl hydrazone
CEF: Cefepime
CFU: Colony-forming unit
CGL: *Citrus grandis*
CHL: Chloramphenicol
CIP: Ciprofloxacin
CMB: *Cucurbita maxima* beans
CPZ: Chlorpromazine
DEB: *Dacryodes edulis* bark
DEL: *Dacryodes edulis* leaves
DES: *Dacryodes edulis* seeds
DMSO: Dimethylsulfoxide
EPI: Efflux pump inhibitors
ERY: Erythromycin
HEL: *Hibiscus esculentus* leaves
IBL: *Ipomoea batatas* leaves
IGB: *Irvingia gabonensis* beans
INT: *p*-Iodonitrotetrazolium chloride
KAN: Kanamycin
MBC: Minimal bactericidal concentration
MDR: Multidrug-resistant
MHB: Mueller Hinton broth
MIC: Minimal inhibitory concentration
MRSA: Methicillin-resistant *Staphylococcus aureus*
PVL: *Phaseolus vulgaris* leaves
RA: Reference antibiotics
RHB: *Ricinodendron heudelotii* bark
RHL: *Ricinodendron heudelotii* leaves
SA: *Staphylococcus aureus*
SML: *Spondias mombin* leaves
SOL: *Saccharum officinarum* leaves
STR: Streptomycin
TCB: *Theobroma cacao* beans
TCL: *Theobroma cacao* leaves
TET: Tetracycline
UGB: *Uapaca guineensis* bark
UGL: *Uapaca guineensis* leaves.
