# Supplementary material for: Antistaphylococcal and Antibiotic Resistance Modulatory Activities of Thirteen Cameroonian Edible Plants against Resistant Phenotypes
Source: Int J Microbiol. 2018 Jul 3;2018:1920198. doi: 10.1155/2018/1920198 (PMC6051259; doi:10.1155/2018/1920198)
Supplement: Supplementary Materials — Preliminary evaluation of antibiotic resistance modulatory activity of extracts against S. aureus SA88 (Table S1); antibiotic resistance modulatory activity of the bark methanol extract from Dacryodes edulis bark (DEB) (Table S2); the leaf methanol extract from Dacryodes edulis leaves (DEL) (Table S3); the seed methanol extract from Dacryodes edulis seeds (DES) (Table S4); the leaf methanol extract from Ricinodendron heudelotii leaves (RHL) (Table S5); and the bark methanol extract from Uapaca guineensis bark (UGB) (Table S6) at their MIC/2 and MIC/4 on selected strains of Staphylococcus aureus. [file 1920198.f1.docx]

**Anti-Staphylococcal and Antibiotic Resistance Modulatory Activities of thirteen Cameroonian Edible Plants against Resistant Phenotypes**

Brice E. N. Wamba^1^, Armelle T. Mbaveng^1^, Paul Nayim^1^, Joachim K. Dzotam^1^, Ornella J.T. Ngalani^1^, and Victor Kuete^1*^

*^1^Department of Biochemistry, Faculty of Science, University of Dschang, Cameroon*

**Author’s addresses**

*Elvis B. N. Wamba:* [*wambaelvis@yahoo.fr*](mailto:wambaelvis@yahoo.fr)

*Armelle T. Mbaveng:* [*armbatsa@yahoo.fr*](mailto:armbatsa@yahoo.fr)

*Paul Nayim:* [*nayimpaul@yahoo.fr*](mailto:nayimpaul@yahoo.fr)

*Joachim K. Dzotam:* [*kamgue_joachim@yahoo.fr*](mailto:kamgue_joachim@yahoo.fr)

*Ornella J.T. Ngalani:* [*ngalaniornella@yahoo.fr*](mailto:ngalaniornella@yahoo.fr)

*Victor Kuete:* [*kuetevictor@yahoo.fr*](mailto:kuetevictor@yahoo.fr)

**Corresponding authors:**

** Tel.: +237 677355927; E-mail address: kuetevictor@yahoo.fr (Prof. Dr Victor Kuete)*

**Table S1. Preliminary evaluation of antibiotic-resistance modulatory activity of selected extracts at sub-inhibitory concentrations against *S. aureus SA88***

| **Plant extracts^a^** | **Extract concentrations** | **MIC of antibiotic (μg/mL) alone and in combination with extracts and fold increase of activity (in bracket)** | | | | | | | |
| --- | --- | --- | --- | --- | --- | --- | --- | --- | --- |
|  |  | **CHL** | **TET** | **CIP** | **AMP** | **CEF** | **ERY** | **STR** | **KAN** |
|  | 0 | 256 | - | 2 | **-** | **-** | 32 | 8 | 4 |
| AIB | MIC/2 | 64 **(4)** | 64 **(>2)** | ≤0.5 **(≥4)** | **-** | **-** | 32 (1) | 4 **(2)** | 4(1) |
|  | MIC/4 | 128 **(2)** | - | 1 **(2)** | **-** | **-** | 64 (0.5) | 4 **(2)** | 4(1) |
|  | MIC/8 | 128 **(2)** | - | 1 **(2)** | **-** | **-** | 64 (0.5) | 8 (1) | 4(1) |
|  | MIC/16 | 128 **(2)** | - | 1 **(2)** | **-** | **-** | 64 (0.5) | 8 (1) | 4(1) |
| DES | MIC/2 | 32 **(8)** | 64 **(>2)** | ≤0.5 **(≥4)** | **-** | 128 **(>2)** | 64 (0.5) | 4 **(2)** | 2 **(2)** |
|  | MIC/4 | 128 **(2)** | 64 **(>2)** | ≤0.5 **(≥4)** | **-** | **-** | 64 (0.5) | 4 **(2)** | 2 **(2)** |
|  | MIC/8 | 128 **(2)** | 64 **(>2)** | 1 **(2)** | **-** | **-** | 64 (0.5) | 8 (1) | 4(1) |
|  | MIC/16 | 128 **(2)** | 64 **(>2)** | 1 **(2)** | **-** | **-** | 64 (0.5) | 8 (1) | 4(1) |
| DEB | MIC/2 | 64 **(4)** | - | 1 **(2)** | **-** | **-** | 32 (1) | 4 **(2)** | 2 **(2)** |
|  | MIC/4 | 128 **(2)** | - | 1 **(2)** | **-** | **-** | 64 (0.5) | 4 **(2)** | 4(1) |
|  | MIC/8 | 128 **(2)** | - | 1 **(2)** | **-** | **-** | 64 (0.5) | 8 (1) | 4(1) |
|  | MIC/16 | 128 **(2)** | - | 1 **(2)** | **-** | **-** | 64 (0.5) | 8 (1) | 4(1) |
| DEL | MIC/2 | 64 **(4)** | 64 **(>2)** | ≤0.5 **(≥4)** | **-** | 128 **(>2)** | 8 **(4)** | ≤2 **(≥4)** | 4(1) |
|  | MIC/4 | 64 **(4)** | 64 **(>2)** | ≤0.5 **(≥4)** | **-** | **-** | 8 **(4)** | ≤2 **(≥4)** | 4(1) |
|  | MIC/8 | 128 **(2)** | **-** | 1 **(2)** | **-** | **-** | 32 (1) | 8 (1) | 4(1) |
|  | MIC/16 | 128 **(2)** | **-** | 2 (1) | **-** | **-** | 32 (1) | 16 (0.5) | 4(1) |
| PVL | MIC/2 | 128 **(2)** | 64 **(>4)** | 2 (1) | **-** | **-** | 128 (0.25) | 16 (0.5) | 8 (0.5) |
|  | MIC/4 | 128 **(2)** | 64**(>4)** | 2 (1) | **-** | **-** | 128 (0.25) | 16 (0.5) | 8 (0.5) |
|  | MIC/8 | 128 **(2)** | - | 2 (1) | **-** | **-** | 128 (0.25) | 16 (0.5) | 8 (0.5) |
|  | MIC/16 | 128 **(2)** | - | 2 (1) | **-** | **-** | 128 (0.25) | 16 (0.5) | 8 (0.5) |
| RHL | MIC/2 | 64 **(4)** | 32 **(>8)** | 1 **(2)** | ≤2 **(≥128)** | ≤2 **(≥128)** | 16 **(2)** | ≤2 **(≥4)** | 2 **(2)** |
|  | MIC/4 | 128 **(2)** | 32 **(>8)** | 1 **(2)** | 256 **(≥2)** | 256 **(≥2)** | 64 (0.5) | 8 (1) | 2 **(2)** |
|  | MIC/8 | 128 **(2)** | - | 2 (1) | **-** | **-** | 64 (0.5) | 8 (1) | 8 (0.5) |
|  | MIC/16 | 128 **(2)** | - | 2 (1) | **-** | **-** | 64 (0.5) | 8 (1) | 8 (0.5) |
| UGB | 128 **(2)** | 64 **(>4)** | 1 **(2)** | ≤2 **(≥128)** | **-** | 8 **(4)** | ≤2 **(≥4)** | 2 **(2)** | 128 **(2)** |
|  | 128 **(2)** | 64 **(>4)** | 1 **(2)** | **-** | **-** | 8 **(4)** | ≤2 **(≥4)** | 2 **(2)** | 128 **(2)** |
|  | 256 (1) | - | 2 (1) | **-** | **-** | 32 (1) | 16 (0.5) | 8 (0.5) | 256 (1) |
|  | 256 (1) | - | 2 (1) | **-** | **-** | 32 (1) | 16 (0.5) | 8 (0.5) | 256 (1) |

^a^Samples [AIB : *Azadirachta indica* bark*,* DES : *Dacryodes edulis* seeds, DEB : *Dacryodes edulis* bark, DEL : *Dacryodes edulis* leaves, *PVL : Phaseolus vulgaris* leaves, RHL: *Ricinodendron heudelottii* leaves, UGB : *Uapaca guineensis* bark]; TET: tetracycline, KAN: kanamycin, ERY: erythromycin, CHL: chloramphenicol, CIP: ciprofloxacin, AMP: ampicillin STR: streptomycine, CEF : cefepime; −: MIC not detected at up to 256 μg/mL; (in bracket): Modulating factor; MIC Minimal Inhibitory Concentration; Values in bold represent modulating factor ≥ 2.

**Table S2.** Resistance-modulating effects of the bark methanol extract from *Dacryodes edulis* (DEB) at its MIC/2 and MIC/4 on selected strains of *Staphyloccus aureus*

| **Antibiotics** | **Extract concentration** | **Bacterial strains, MIC (μg/mL) of antibiotics in the absence and presence (in bracket) of the extract (in bracket)** | | | | | | | | | **Antibiotic-modulating effect (%)** |
| --- | --- | --- | --- | --- | --- | --- | --- | --- | --- | --- | --- |
|  |  | **MRSA3** | **MRSA4** | **MRSA9** | **MRSA11** | **MRSA12** | **ATCC 25923** | **SA18** | **SA36** | **SA64** |  |
| CIP | 0 | ≤ 0.5 | 8 | ≤ 0.5 | 1 | 2 | 8 | 2 | 4 | 8 |  |
|  | MIC/2 | ≤ 0.5 (na) | 8 (1) | ≤ 0.5 (na) | 1 (1) | 2 (1) | 8 (1) | ≤0.5 **(≥4)** | ≤ 2 **(≥2)** | ≤0.5 **(≥16)** | 33.33 |
|  | MIC/4 | ≤ 0.5 (na) | 8 (1) | ≤ 0.5 (na) | 1 (1) | 2 (1) | 8 (1) | ≤0.5 **(≥4)** | ≤ 2 **(≥2)** | ≤0.5 **(≥16)** | 33.33 |
| CHL | 0 | 64 | 4 | 128 | 128 | 128 | 64 | 8 | 128 | 32 |  |
|  | MIC/2 | 64 (1) | ≤ 2 **(≥2)** | 64 **(2)** | 128 (1) | 64 **(2)** | 32 **(2)** | ≤ 2 **(≥4)** | 64 **(2)** | ≤2 **(≥16)** | **77.78** |
|  | MIC/4 | 64 (1) | 4 (1) | 64 **(2)** | 128 (1) | 64 **(2)** | 32 **(2)** | 4 **(2)** | 128 (1) | ≤2 **(≥16)** | 55.56 |
| TET | 0 | 32 | 2 | 32 | 64 | 64 | ≤0.5 | 2 | 1 | 1 |  |
|  | MIC/2 | 16 **(2)** | ≤0,5 **(≥4)** | 16 **(2)** | 32 **(2)** | 32 **(2)** | ≤0.5 (na) | 1 **(2)** | ≤0.5 (≥1) | ≤0.5 (≥1) | 66.67 |
|  | MIC/4 | 16 **(2)** | ≤0,5 **(≥4)** | 16 **(2)** | 32 **(2)** | 32 **(2)** | ≤0.5 (na) | 2(1) | ≤0.5 (≥1) | ≤0.5 (≥1) | 55.56 |
| ERY | 0 | 32 | 16 | 8 | 16 | 16 | 16 | 32 | ≤ 2 | 8 |  |
|  | MIC/2 | 8 **(4)** | ≤ 2 **(≥8)** | 8 (1) | 4 **(4)** | 8 **(2)** | ≤ 2 **(≥8)** | 32 (1) | ≤ 2 (na) | ≤ 2 **(≥4)** | 55.56 |
|  | MIC/4 | 8 **(4)** | ≤ 2 **(≥8)** | 8 (1) | 4 **(4)** | 8 **(2)** | ≤ 2 **(≥8)** | 32 (1) | ≤ 2 (na) | ≤ 2 **(≥4)** | 66.67 |
| KAN | 0 | 8 | 256 | 16 | 16 | 64 | ≤ 2 | 8 | ≤ 2 | 32 |  |
|  | MIC/2 | 8 (1) | 256 (1) | 16 (1) | 16 (1) | 128 (0.5) | ≤ 2 (na) | ≤ 2 **(≥4)** | ≤ 2 (1) | ≤ 2 **(≥16)** | 11.11 |
|  | MIC/4 | 8 (1) | 256 (1) | 16 (1) | 16 (1) | 256 (0.25) | ≤ 2 (na) | ≤ 2 **(≥4)** | ≤ 2 (1) | ≤ 2 **(≥16)** | 11.11 |
| CEF | 0 | - | - | - | - | - | - | - | 32 | 128 |  |
|  | MIC/2 | - | - | - | - | - | - | - | 8 **(4)** | ≤2 **(≥64)** | 22.22 |
|  | MIC/4 | - | - | - | - | - | - | - | 8 **(4)** | ≤2 **(≥64)** | 22.22 |
| STR | 0 | 128 | 64 | 128 | 64 | 64 | 256 | 128 | 128 | 16 |  |
|  | MIC/2 | 64 **(2)** | 4 **(16)** | 64 **(2)** | 64 (1) | 64 (1) | 256 (1) | 64 **(2)** | 64 **(2)** | ≤ 2 **(≥8)** | 66.67 |
|  | MIC/4 | 64 **(2)** | 64 (1) | 64 **(2)** | 64 (1) | 64 (1) | 256 (1) | 64 **(2)** | 128 (1) | ≤ 2 **(≥8)** | 44.44 |
| AMP | 0 | - | 256 | - | - | - | - | 128 | 256 | - |  |
|  | MIC/2 | - | 256 (1) | - | - | - | - | 128 (1) | 256 (1) | - | 0 |
|  | MIC/4 | - | 256 (1) | - | - | - | - | 128 (1) | 256 (1) | - | 0 |

TET: tetracycline, KAN: kanamycin, ERY: erythromycin, CHL: chloramphenicol, CIP: ciprofloxacin, AMP: ampicillin STR: streptomycine, CEF : cefepime; −: MIC not detected at up to 256 μg/mL; (): Modulating factor; na: not applicable ; MIC Minimal Inhibitory Concentration ; Percentage of antibiotic’s modulating effect by the plant extracts; Values in bold represent modulating factor ≥ 2 and modulating effect observed on more than 70% of the tested MDR bacteria.

**Table S3.** Resistance-modulating effects of the leave methanol extract from *Dacryodes edulis* (DEL) at its MIC/2 and MIC/4 on selected strains of *Staphyloccus aureus*

| **Antibiotics** | **Extract concentration** | **Bacterial strains, MIC (μg/mL) of antibiotics in the absence and presence (in bracket) of the extract (in bracket)** | | | | | | | | | **Antibiotic-modulating effect (%)** |
| --- | --- | --- | --- | --- | --- | --- | --- | --- | --- | --- | --- |
|  |  | **MRSA3** | **MRSA4** | **MRSA9** | **MRSA11** | **MRSA12** | **ATCC 25923** | **SA18** | **SA36** | **SA64** |  |
| CIP | 0 | ≤ 0.5 | 8 | ≤ 0.5 | 1 | 2 | 8 | 2 | 4 | 8 |  |
|  | MIC/2 | ≤ 0.5 (na) | 4 **(2)** | ≤ 0.5 (na) | ≤ 0.5 **(≥2)** | 2 (1) | 4 **(2)** | ≤ 0.5 **(≥4)** | ≤ 0.5 **(≥8)** | 1 **(8)** | 66.67 |
|  | MIC/4 | ≤ 0.5 (na) | 8 (1) | ≤ 0.5 (na) | ≤ 0.5 **(≥2)** | 2 (1) | 8 (1) | ≤ 0.5 **(≥4)** | ≤ 0.5 **(≥8)** | 1 **(8)** | 44.44 |
| CHL | 0 | 64 | 4 | 128 | 128 | 128 | 64 | 8 | 128 | 32 |  |
|  | MIC/2 | 32 **(2)** | ≤ 2 **(≥2)** | 64 **(2)** | 128 (1) | 64 (1) | 32 **(2)** | ≤ 2 **(≥4)** | 64 **(2)** | ≤ 2 **(≥16)** | **77.78** |
|  | MIC/4 | 64 (1) | 4 (1) | 64 **(2)** | 128 (1) | 64 (1) | 32 **(2)** | 4 **(2)** | 64 **(2)** | ≤ 2 **(≥16)** | 55.56 |
| TET | 0 | 32 | 2 | 32 | 64 | 64 | ≤0.5 | 2 | 1 | 1 |  |
|  | MIC/2 | 32 (1) | ≤ 0.5 | 32 (1) | 32 **(2)** | 32 **(2)** | ≤ 0.5 (na) | 1 **(2)** | ≤ 0.5 **(≥2)** | ≤ 0.5 **(≥2)** | 55.56 |
|  | MIC/4 | 32 (1) | ≤ 0.5 | 32 (1) | 32 **(2)** | 32 **(2)** | ≤ 0.5 (na) | 1 **(2)** | 1 (1) | ≤ 0.5 **(≥2)** | 44.44 |
| ERY | 0 | 32 | 16 | 8 | 16 | 16 | 16 | 32 | ≤ 2 | 8 |  |
|  | MIC/2 | 16 **(2)** | 4 **(4)** | 8 (1) | 8 **(2)** | 16 (1) | ≤ 2 **(≥8)** | ≤ 2 **(≥16)** | ≤ 2 (na) | ≤ 2 **(≥4)** | 66.67 |
|  | MIC/4 | 16 **(2)** | 4 **(4)** | 8 (1) | 8 **(2)** | 16 (1) | ≤ 2 **(≥8)** | ≤ 2**(≥16)** | ≤ 2 (na) | ≤ 2 **(≥4)** | 66.67 |
| KAN | 0 | 8 | 256 | 16 | 16 | 64 | ≤ 2 | 8 | ≤ 2 | 32 |  |
|  | MIC/2 | 4 **(2)** | 256 (1) | 16 (1) | 8 **(2)** | 64 (1) | ≤ 2 (na) | ≤ 2 **(≥4)** | ≤ 2 (na) | ≤ 2 **(≥16)** | 44.44 |
|  | MIC/4 | 8 (1) | 256 (1) | 16 (1) | 8 **(2)** | 64 (1) | ≤ 2 (na) | ≤ 2 **(≥4)** | ≤ 2 (na) | ≤ 2 **(≥16)** | 33.33 |
| CEF | 0 | - | - | - | - | - | - | - | 32 | 128 |  |
|  | MIC/2 | - | - | - | - | - | - | - | 4 **(8)** | 8 **(16)** | 22.22 |
|  | MIC/4 | - | - | - | - | - | - | - | 16 **(2)** | 64 **(2)** | 22.22 |
| STR | 0 | 128 | 64 | 128 | 64 | 64 | 256 | 128 | 128 | 16 |  |
|  | MIC/2 | 64 **(2)** | ≤ 2 **(≥32)** | 64 **(2)** | 64 (1) | 64 (1) | 128 **(2)** | 64 **(2)** | ≤ 2 **(≥64)** | ≤ 2 **(≥8)** | **77.78** |
|  | MIC/4 | 64 **(2)** | 64 (1) | 64 **(2)** | 64 (1) | 64 (1) | 256 (1) | 64 **(2)** | 128 (1) | ≤ 2 **(≥8)** | 44.44 |
| AMP | 0 | - | 256 | - | - | - | - | 128 | 256 | - |  |
|  | MIC/2 | - | 256 (1) | - | - | - | - | 128 (1) | ≤ 2 **(≥128)** | - | 11.11 |
|  | MIC/4 | - | 256 (1) | - | - | - | - | 128 (1) | 256 (1) | - | 0 |

TET: tetracycline, KAN: kanamycin, ERY: erythromycin, CHL: chloramphenicol, CIP: ciprofloxacin, AMP: ampicillin STR: streptomycine, CEF : cefepime; −: MIC not detected at up to 256 μg/mL; (): Modulating factor; na: not applicable ; MIC Minimal Inhibitory Concentration ; Percentage of antibiotic’s modulating effect by the plant extracts; Values in bold represent modulating factor ≥ 2 and modulating effect observed on more than 70% of the tested MDR bacteria.

**Table S4.** Resistance modulating effects of the seeds methanol extract from *Dacryodes edulis* (DES) at its MIC/2 and MIC/4 on selected strains of *Staphyloccus aureus*

| **Antibiotics** | **Extract concentration** | **Bacterial strains, MIC (μg/mL) of antibiotics in the absence and presence (in bracket) of the extract (in bracket)** | | | | | | | | | **Antibiotic-modulating effect (%)** |
| --- | --- | --- | --- | --- | --- | --- | --- | --- | --- | --- | --- |
|  |  | **MRSA3** | **MRSA4** | **MRSA9** | **MRSA11** | **MRSA12** | **ATCC 25923** | **SA18** | **SA36** | **SA64** |  |
| CIP | 0 | ≤0.5 | 8 | 8 | 2 | 2 | 8 | 32 | 16 | 8 |  |
|  | MIC/2 | ≤0.5 (na) | ≤0.5 **(≥16)** | 8 (1) | ≤0.5 **(≥4)** | ≤0.5 **(≥4)** | 4 **(2)** | ≤0.5 **(≥64)** | ≤0.5 **(≥32)** | ≤0.5 **(≥16)** | **77.78** |
|  | MIC/4 | ≤0.5 (na) | ≤0.5 **(≥16)** | 8 (1) | ≤0.5 **(≥4)** | ≤0.5 **(≥4)** | 8 (1) | ≤0.5 **(≥64)** | ≤0.5 **(≥32)** | ≤0.5 **(≥16)** | 66.67 |
| CHL | 0 | 16 | 128 | 128 | 128 | 128 | 32 | 16 | 32 | 32 |  |
|  | MIC/2 | ≤2 **(≥8)** | 8 **(16)** | 64 **(2)** | 4 **(32)** | 32 **(4)** | 8 **(4)** | ≤2 **(≥8)** | 8 **(4)** | ≤2 **(≥16)** | **100** |
|  | MIC/4 | ≤2 **(≥8)** | 8 **(16)** | 64 **(2)** | 4 **(32)** | 32 **(4)** | 32 **(4)** | 16 (1) | 16 **(2)** | 8 **(4)** | **88.89** |
| TET | 0 | 32 | 2 | 32 | 32 | 32 | ≤0.5 | 16 | 2 | 16 |  |
|  | MIC/2 | ≤0.5 **(≥64)** | 2 (1) | 16 **(2)** | 8 **(4)** | 16 **(2)** | ≤0.5 (na) | ≤0.5 **(≥32)** | ≤0.5 **(≥4)** | ≤0.5 **(≥32)** | **77.78** |
|  | MIC/4 | ≤0.5 **(≥64)** | 2 (1) | 16 **(2)** | 8 **(4)** | 16 **(2)** | ≤0.5 (na) | ≤0.5 **(≥32)** | ≤0.5 **(≥4)** | ≤0.5 **(≥32)** | **77.78** |
| ERY | 0 | 16 | 128 | 128 | 32 | 64 | ≤2 | 64 | 64 | 16 |  |
|  | MIC/2 | ≤2 **(≥8)** | 128 (1) | 128 (1) | 8 **(4)** | 4 **(16)** | ≤2 (na) | ≤2 **(≥32)** | 4 **(16)** | ≤2 **(≥8)** | 66.67 |
|  | MIC/4 | 8 **(2)** | 128 (1) | 128 (1) | 8 **(4)** | 4 **(16)** | ≤2 (na) | ≤2 **(≥32)** | 4 **(16)** | ≤2 **(≥8)** | 66.67 |
| KAN | 0 | 16 | 256 | 128 | 16 | 16 | ≤2 | 64 | 4 | 32 |  |
|  | MIC/2 | 16 (1) | 256 (1) | 32 **(4)** | ≤2 **(≥8)** | 4 **(4)** | ≤2 (na) | ≤2 **(≥32)** | ≤2 **(≥2)** | ≤2 **(≥16)** | 66.67 |
|  | MIC/4 | 16 (1) | 256 (1) | 32 **(4)** | ≤2 **(≥8)** | 4 **(4)** | ≤2 (na) | ≤2 **(≥32)** | ≤2 **(≥2)** | ≤2 **(≥16)** | 66.67 |
| CEF | 0 | - | - | - | - | - | - | - | 16 | 64 |  |
|  | MIC/2 | - | - | - | - | - | - | 64 **(>4)** | ≤2 **(≥8)** | 8 **(8)** | 33.33 |
|  | MIC/4 | - | - | - | - | - | - | 64 **(>4)** | 8 **(2)** | 64 (1) | 22.22 |
| STR | 0 | 64 | 128 | 32 | 32 | 256 | - | 256 | 8 | 16 |  |
|  | MIC/2 | ≤2 **(≥32)** | 4 **(32)** | 32 (1) | 4 **(8)** | 64 **(4)** | 128 **(>2)** | 4 **(64)** | ≤2 **(≥4)** | ≤2 **(≥8)** | **88.89** |
|  | MIC/4 | ≤2 **(≥32)** | 128 (1) | 32 (1) | 4 **(8)** | 64 **(4)** | 128 **(>2)** | 4 **(64)** | ≤2 **(≥4)** | ≤2 **(≥8)** | **77.78** |
| AMP | 0 | - | - | - | - | - | - | - | 128 | 16 |  |
|  | MIC/2 | - | - | - | - | - | - | 64 **(>4)** | ≤2 **(≥64)** | ≤2 **(≥8)** | 33.33 |
|  | MIC/4 | - | - | - | - | - | - | 128 **(>2)** | ≤2 **(≥64)** | ≤2 **(≥8)** | 33.33 |

TET: tetracycline, KAN: kanamycin, ERY: erythromycin, CHL: chloramphenicol, CIP: ciprofloxacin, AMP: ampicillin STR: streptomycine, CEF : cefepime; −: MIC not detected at up to 256 μg/mL; (): Modulating factor; na: not applicable ; MIC Minimal Inhibitory Concentration ; Percentage of antibiotic’s modulating effect by the plant extracts; Values in bold represent modulating factor ≥ 2 and modulating effect observed on more than 70% of the tested MDR bacteria.

**Table S5.** Resistance modulating effects of the leaves methanol extract from *Ricinodendron heudelottii* (RHL) at its MIC/2 and MIC/4 on selected strains of *Staphyloccus aureus*

| **Antibiotics** | **Extract concentration** | **Bacterial strains, MIC (μg/mL) of antibiotics in the absence and presence of the extract (in bracket)** | | | | | | | | | **Antibiotic-modulating effect (%)** |
| --- | --- | --- | --- | --- | --- | --- | --- | --- | --- | --- | --- |
|  |  | **MRSA3** | **MRSA4** | **MRSA9** | **MRSA11** | **MRSA12** | **ATCC 25923** | **SA18** | **SA36** | **SA64** |  |
| CIP | 0 | ≤0.5 | 8 | 8 | 2 | 2 | 8 | 32 | 16 | 8 |  |
|  | MIC/2 | ≤0.5 (na) | ≤0.5 **(≥16)** | ≤0.5 **(≥16)** | ≤0.5 **(≥4)** | ≤0.5 **(≥4)** | 4 **(2)** | ≤0.5 **(≥64)** | ≤0.5 **(≥32)** | ≤0.5 **(≥16)** | **88.89** |
|  | MIC/4 | ≤0.5 (na) | ≤0.5 **(≥16)** | 4 **(2)** | ≤0.5 **(≥4)** | ≤0.5 **(≥4)** | 8 (1) | ≤0.5 **(≥64)** | ≤0.5 **(≥32)** | ≤0.5 **(≥16)** | **77.78** |
| CHL | 0 | 16 | 128 | 128 | 128 | 128 | 32 | 16 | 32 | 32 |  |
|  | MIC/2 | 8 **(2)** | 4 **(32)** | 64 **(2)** | ≤2 **(≥256)** | 64 **(2)** | 32 (1) | ≤2 **(≥8)** | ≤2 **(≥16)** | ≤2 **(≥16)** | **88.89** |
|  | MIC/4 | 8 **(2)** | 4 **(32)** | 64 **(2)** | ≤2 **(≥256)** | 64 **(2)** | 32 (1) | ≤2 **(≥8)** | ≤2 **(≥16)** | ≤2 **(≥16)** | **88.89** |
| TET | 0 | 32 | 2 | 32 | 32 | 32 | ≤0.5 | 16 | 2 | 16 |  |
|  | MIC/2 | 2 **(16)** | 2 (1) | 16 **(2)** | 8 **(4)** | ≤0.5 **(≥64)** | ≤0.5 (na) | ≤0.5 **(≥32)** | ≤0.5 **(≥4)** | ≤0.5 **(≥32)** | **77.78** |
|  | MIC/4 | 2 **(16)** | 2 (1) | 16 **(2)** | 8 **(4)** | ≤0.5 **(≥64)** | ≤0.5 (na) | ≤0.5 **(≥32)** | ≤0.5 **(≥4)** | ≤0.5 **(≥32)** | **77.78** |
| ERY | 0 | 16 | 128 | 128 | 32 | 64 | ≤2 | 64 | 64 | 16 |  |
|  | MIC/2 | ≤2 **(≥32)** | ≤2 **(≥256)** | 8 **(16)** | 8 **(4)** | 8 **(8)** | ≤2 (na) | 8 **(8)** | ≤2 **(≥128)** | ≤2 **(≥32)** | **88.89** |
|  | MIC/4 | ≤2 **(≥32)** | ≤2 **(≥256)** | 128 (1) | 8 **(4)** | 32 **(2)** | ≤2 (na) | 8 **(8)** | ≤2 **(≥128)** | ≤2 **(≥32)** | **77.78** |
| KAN | 0 | 16 | 256 | 128 | 16 | 16 | ≤2 | 64 | 4 | 32 |  |
|  | MIC/2 | ≤2 **(≥8)** | 4 **(64)** | 8 **(16)** | ≤2 **(≥8)** | ≤2 **(≥8)** | ≤2 (na) | ≤2 **(≥32)** | ≤2 **(≥2)** | ≤2 **(≥16)** | **88.89** |
|  | MIC/4 | ≤2 **(≥8)** | 256 (1) | 8 **(16)** | ≤2 **(≥8)** | ≤2 **(≥8)** | ≤2 (na) | ≤2 **(≥32)** | ≤2 **(≥2)** | ≤2 **(≥16)** | **77.78** |
| CEF | 0 | - | - | - | - | - | - | - | 16 | 64 |  |
|  | MIC/2 | - | - | - | - | - | - | 128 **(>2)** | 4 **(4)** | ≤2 **(≥32)** | 33.33 |
|  | MIC/4 | - | - | - | - | - | - | 128 **(>2)** | 4 **(4)** | ≤2 **(≥32)** | 33.33 |
| STR | 0 | 64 | 128 | 32 | 32 | 256 | - | 256 | 8 | 16 |  |
|  | MIC/2 | 16 **(4)** | ≤2 **(≥64)** | ≤2 **(≥16)** | 8 **(4)** | 32 **(8)** | - | ≤2 **(≥128)** | ≤2 **(≥4)** | ≤2 **(≥8)** | **88.89** |
|  | MIC/4 | 16 **(4)** | 128 (1) | 32 (1) | 8 **(4)** | 32 **(8)** | - | 4 **(64)** | ≤2 **(≥4)** | ≤2 **(≥8)** | 66.67 |
| AMP | 0 | - | - | - | - | - | - | - | 128 | 16 |  |
|  | MIC/2 | - | - | - | - | - | - | 16 **(≥16)** | ≤2 **(≥64)** | 4 **(4)** | 33.33 |
|  | MIC/4 | - | - | - | - | - | - | 16 **(≥16)** | 64 **(2)** | 8 **(2)** | 33.33 |

TET: tetracycline, KAN: kanamycin, ERY: erythromycin, CHL: chloramphenicol, CIP: ciprofloxacin, AMP: ampicillin STR: streptomycine, CEF : cefepime; −: MIC not detected at up to 256 μg/mL; (): Modulating factor; na: not applicable ; MIC Minimal Inhibitory Concentration ; Percentage of antibiotic’s modulating effect by the plant extracts; Values in bold represent modulating factor ≥ 2 and modulating effect observed on more than 70% of the tested MDR bacteria.

**Table S6.** Resistance-modulating effects of the bark methanol extract from *Uapaca guineensis* (UGB) at its MIC/2 and MIC/4 on selected strains of *Staphyloccus aureus*

| **Antibiotics** | **Extract concentration** | **Bacterial strains, MIC (μg/mL) of antibiotics in the absence and presence of the extract(in bracket)** | | | | | | | | | **Antibiotic-modulating effect (%)** |
| --- | --- | --- | --- | --- | --- | --- | --- | --- | --- | --- | --- |
|  |  | **MRSA3** | **MRSA4** | **MRSA9** | **MRSA11** | **MRSA12** | **ATCC 25923** | **SA18** | **SA36** | **SA64** |  |
| CIP | 0 | ≤0.5 | 8 | 8 | 2 | 2 | 8 | 32 | 16 | 8 |  |
|  | MIC/2 | ≤0.5 (na) | 8 (1) | 8 (1) | ≤0.5 **(≥4)** | ≤0.5 **(≥4)** | 4 **(2)** | ≤0.5 **(≥64)** | ≤0.5 **(≥32)** | ≤0.5 **(≥16)** | 66.67 |
|  | MIC/4 | ≤0.5 (na) | 8 (1) | 8 (1) | ≤0.5 **(≥4)** | ≤0.5 **(≥4)** | 4 **(2)** | ≤0.5 **(≥64)** | ≤0.5 **(≥32)** | ≤0.5 **(≥16)** | 66.67 |
| CHL | 0 | 16 | 128 | 128 | 128 | 128 | 32 | 16 | 32 | 32 |  |
|  | MIC/2 | 16 (1) | 4 **(32)** | 64 **(2)** | 4 **(32)** | 64 **(2)** | 32 (1) | ≤2 **(≥8)** | 4 **(8)** | ≤2 **(≥16)** | **77.78** |
|  | MIC/4 | 16 (1) | 32 **(4)** | 64 **(2)** | 4 **(32)** | 64 **(2)** | 32 (1) | ≤2 **(≥8)** | 4 **(8)** | ≤2 **(≥16)** | **77.78** |
| TET | 0 | 32 | 2 | 32 | 32 | 32 | ≤0.5 | 16 | 2 | 16 |  |
|  | MIC/2 | 32 (1) | 2 (1) | 32 (1) | 16 **(2)** | 32 (1) | ≤0.5 (na) | ≤0.5 **(≥32)** | ≤0.5 **(≥4)** | ≤0.5 **(≥32)** | 44.44 |
|  | MIC/4 | 32 (1) | 2 (1) | 32 (1) | 16 **(2)** | 32 (1) | ≤0.5 (na) | ≤0.5 **(≥32)** | ≤0.5 **(≥4)** | ≤0.5 **(≥32)** | 44.44 |
| ERY | 0 | 16 | 128 | 128 | 32 | 64 | ≤2 | 64 | 64 | 16 |  |
|  | MIC/2 | 16 (1) | 128 (1) | 128 **(8)** | 8 **(4)** | 4 **(16)** | ≤2 (na) | 8 **(8)** | 16 **(4)** | ≤2 **(≥8)** | 66.67 |
|  | MIC/4 | 16 (1) | 128 (1) | 128 **(8)** | 8 **(4)** | 64 (1) | ≤2 (na) | 8 **(8)** | 16 **(4)** | 4 **(4)** | 55.56 |
| KAN | 0 | 16 | 256 | 128 | 16 | 16 | ≤2 | 64 | 4 | 32 |  |
|  | MIC/2 | 8 **(2)** | 256 (1) | 16 **(8)** | 8 **(2)** | 8 **(2)** | ≤2 (na) | ≤2 **(≥32)** | ≤2 **(≥2)** | ≤2 **(≥16)** | **77.78** |
|  | MIC/4 | 8 **(2)** | 256 (1) | 16 **(8)** | 8 **(2)** | 8 **(2)** | ≤2 (na) | ≤2 **(≥32)** | ≤2 **(≥2)** | ≤2 **(≥16)** | **77.78** |
| CEF | 0 | - | - | - | - | - | - | - | 16 | 64 |  |
|  | MIC/2 | - | - | - | - | - | - | 64 **(≥4)** | 8 **(2)** | ≤2 **(≥32)** | 33.33 |
|  | MIC/4 | - | - | - | - | - | - | 64 **(≥4)** | 8 **(2)** | ≤2 **(≥32)** | 33.33 |
| STR | 0 | 64 | 128 | 32 | 32 | 256 | - | 256 | 8 | 16 |  |
|  | MIC/2 | 16 **(4)** | 128 (1) | 32 (1) | 16 **(2)** | 64 **(4)** | 128 **(>2)** | ≤2 **(≥128)** | 4 **(2)** | ≤2 **(≥8)** | **77.78** |
|  | MIC/4 | 16 **(4)** | 128 (1) | 32 (1) | 16 **(2)** | 64 **(4)** | 128 **(>2)** | ≤2 **(≥128)** | 4 **(2)** | ≤2 **(≥8)** | **77.78** |
| AMP | 0 | - | - | - | - | - | - | - | 128 | 16 |  |
|  | MIC/2 | - | - | - | - | - | - | ≤2 **(>128)** | 8 **(16)** | ≤2 **(≥8)** | 33.33 |
|  | MIC/4 | - | - | - | - | - | - | 64 **(>4)** | 8 **(16)** | ≤2 **(≥8)** | 33.33 |

TET: tetracycline, KAN: kanamycin, ERY: erythromycin, CHL: chloramphenicol, CIP: ciprofloxacin, AMP: ampicillin STR: streptomycine, CEF : cefepime; −: MIC not detected at up to 256 μg/mL; (): Modulating factor; na: not applicable ; MIC Minimal Inhibitory Concentration ; Percentage of antibiotic’s modulating effect by the plant extracts; Values in bold represent modulating factor ≥ 2 and modulating effect observed on more than 70% of the tested MDR bacteria.
